# When the whole is greater than the sum of its parts: why machine learning and conventional statistics are complementary for predicting future health outcomes

**DOI:** 10.1093/ckj/sfaf059

**Published:** 2025-02-20

**Authors:** Roemer J Janse, Ameen Abu-Hanna, Iacopo Vagliano, Vianda S Stel, Kitty J Jager, Giovanni Tripepi, Carmine Zoccali, Friedo W Dekker, Merel van Diepen

**Affiliations:** Department of Clinical Epidemiology, Leiden University Medical Center, Leiden, the Netherlands; Department of Medical Informatics, Amsterdam UMC location University of Amsterdam, Amsterdam, the Netherlands; Amsterdam Public Health Research Institute, Methodology, Amsterdam, the Netherlands; Department of Medical Informatics, Amsterdam UMC location University of Amsterdam, Amsterdam, the Netherlands; Amsterdam Public Health Research Institute, Methodology, Amsterdam, the Netherlands; ERA Registry, Department of Medical Informatics, Amsterdam UMC location University of Amsterdam, Amsterdam, the Netherlands; Amsterdam Public Health Research Institute, Quality of Care, Amsterdam, the Netherlands; ERA Registry, Department of Medical Informatics, Amsterdam UMC location University of Amsterdam, Amsterdam, the Netherlands; Amsterdam Public Health Research Institute, Quality of Care, Amsterdam, the Netherlands; CNR-IFC, Clinical Epidemiology and Physiopathology of Renal Diseases and Hypertension, Reggio Calabria, Italy; CNR-IFC, Clinical Epidemiology and Physiopathology of Renal Diseases and Hypertension, Reggio Calabria, Italy; Department of Clinical Epidemiology, Leiden University Medical Center, Leiden, the Netherlands; Department of Clinical Epidemiology, Leiden University Medical Center, Leiden, the Netherlands

**Keywords:** machine learning, prediction, prognosis, regression analysis, statistics

## Abstract

An artificial intelligence boom is currently ongoing, mainly due to large language models, leading to significant interest in artificial intelligence and subsequently also in machine learning (ML). One area where ML is often applied, prediction modelling, has also long been a focus of conventional statistics. As a result, multiple studies have aimed to prove superiority of one of the two scientific disciplines over the other. However, we argue that ML and conventional statistics should not be competing fields. Instead, both fields are intertwined and complementary to each other. To illustrate this, we discuss some essentials of prediction modelling, elaborate on prediction modelling using techniques from conventional statistics, and explain prediction modelling using common ML techniques such as support vector machines, random forests, and artificial neural networks. We then showcase that conventional statistics and ML are in fact similar in many aspects, including underlying statistical concepts and methods used in model development and validation. Finally, we argue that conventional statistics and ML can and should be seen as a single integrated field. This integration can further improve prediction modelling for both disciplines (e.g. regarding fairness and reporting standards) and will support the ultimate goal: developing the best performing prediction models for the patient and healthcare provider.

## BACKGROUND

In 1909, Edward Forster wrote the short story *The Machine Stops*, which is famous for accurately predicting the future [1]. In this story, an omnipotent machine serves the human race, among whom also Vashti and her son Kuno. In the story, Kuno berates his mother: ‘You talk as if a god had made the Machine. […] I believe that you pray to it when you are unhappy. Men made it, do not forget that. Great men, but men. The Machine is much, but not everything.’ These words urge for caution when it comes to the Machine, but they may be remembered for any technological boom.

**Figure 1:**
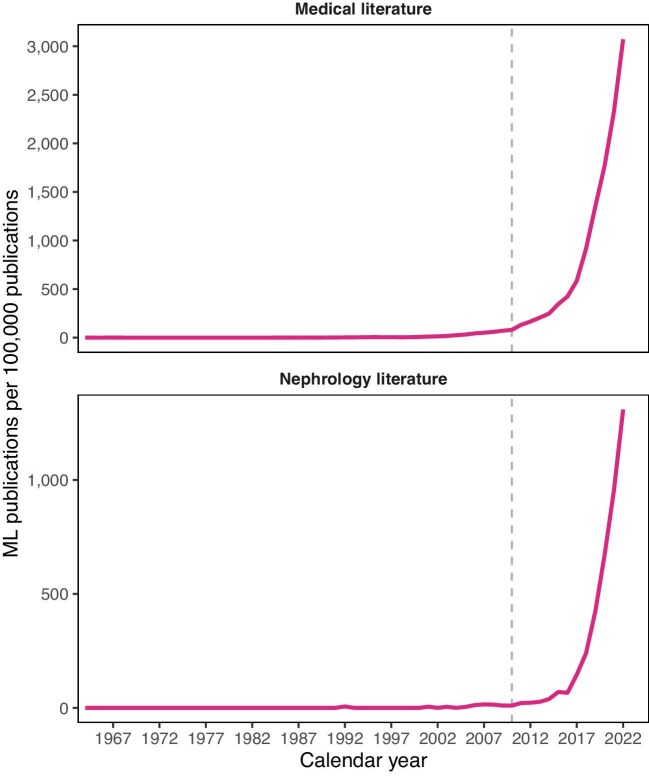
The number of studies mentioning ML published in PubMed per 100 000 total publications at each year until 2022. The dashed line indicates the start of the ongoing AI boom. ML studies were identified with the search query (‘machine learning’[MeSH] OR ‘machine learning’[tiab]. Nephrology studies were identified with the search query (‘kidney’[Mesh] OR ‘renal’[Mesh] OR ‘nephrology’[Mesh] OR ‘renal dialysis’[Mesh] OR ‘kidney’[tiab] OR ‘renal’[tiab] OR ‘nephrology’[tiab] OR ‘dialysis’[tiab].

Since the 2010s, an artificial intelligence (AI) boom has led to a rapid increase in the public uptake of AI [2]. AI is further described and critically reviewed in other papers in the *Clinical Kidney Journal* [3, 4]. Although this boom occurred as a result of deep learning, especially in the form of large language models, it has also led to a rise in the popularity of machine learning (ML, a branch of AI) (Fig. 1), including for developing prediction models [5]. Prediction models are algorithms that give an individual estimate (most often a risk expressed as a probability) to a patient based on a set of relevant predictor variables. Their complexity may range from a weighted checklist of comorbidities (e.g. the Charlson Comorbidity Index to predict mortality) [6] to simulating brain structure (e.g. an artificial neural network [ANN] to predict 6-month primary patency in arteriovenous fistula patients) [7].

Besides ML, conventional statistics (i.e. regression models) have long been used to develop prediction models. Techniques used in conventional statistics also offer reliable and accurate models, with two major examples in the field of nephrology being the Kidney Donor Risk Index for recipient and graft survival after kidney transplantation [8] and the Kidney Failure Risk Equation (KFRE) for the 2- and 5-year risk of kidney failure [9].

With both conventional statistics and ML being applied for prediction modelling and with the hype around ML, many studies have compared ML with conventional statistics [10, 11, 12]. Nonetheless, we must remember that ‘The Machine is much, but not everything’. There is no single best discipline or technique. In fact, conventional statistics and ML partially overlap, albeit with different vocabulary (Table 1), and are complementary to each other, each with their own advantages and disadvantages [13, 14]. In this paper, we introduce some general prediction modelling essentials, conventional statistics (Part I) and ML (Part II) for prediction modelling, highlight their similarities (the sum), and emphasize how they may complement each other (the whole) for the ultimate goal: accurate predictions to inform patients and healthcare providers.

**Table 1:** Different words have the same meaning between the fields of conventional statistics and ML.

Conventional statistics	Machine learning	General meaning
Predictor	Feature	A variable that is used to make a prediction
Outcome	Label	What should be predicted
Estimation	Learning	Determining the parameters of the prediction model based on the data
Development data	Training + validation data	Data on which the prediction model is developed
Validation data	Test data	Data independent of the data on which the prediction model is developed to assess performance
Contingency table	Confusion matrix	A crosstabulation of observed and predicted outcomes showing true positives, true negatives, false positives, and false negatives

## PREDICTION MODELLING ESSENTIALS

Some basic principles always apply to clinical prediction models (hereafter prediction models in short). First and foremost, prediction models should be clinically relevant with a clear definition of the initial timepoint for their application (time zero), in what population they should be used, and for what purpose they should be used.

Second, candidate predictors for the prediction model should be selected by means of literature, clinical availability, and clinical expertise. The number of candidate predictors may be limited by the size of the available data, for which sample size calculations for certain techniques are available [15, 16, 17]. The set of candidate predictors can be further reduced to a number of final predictors using data-driven methods (e.g. stepwise selection), although this probably leads to overfitting [18]: by selecting the best predictors in the development data, predictors reflecting details only present in the development data and not usually in the target population might be selected. In some settings (e.g. omics), the number of available predictors will be too large or the role of different predictors is not clearly known, in which reliance on data-driven selection is unavoidable.

Third, performance of prediction models should be assessed by calibration and discrimination: calibration indicates how close the predictions are to the actual observation (or frequency thereof for binary outcomes) [19]. This is often reported with the calibration slope, calibration-in-the-large, and a calibration plot. Discrimination indicates how well a model can distinguish between individuals with and without the outcome of interest [19] and is often calculated using a concordance statistic (*C*-statistic), which also has extensions to survival and competing risk settings [20, 21, 22]. The *C*-statistic for binary outcomes is mathematically equivalent to the area under the receiver operating characteristic curve (AUC or AUROC). Although both calibration and discrimination are equally important, calibration remains underreported and underrecognized [23]. A model that discriminates well but is poorly calibrated should not be used to either inform or direct clinical practice.

**Figure 2:**
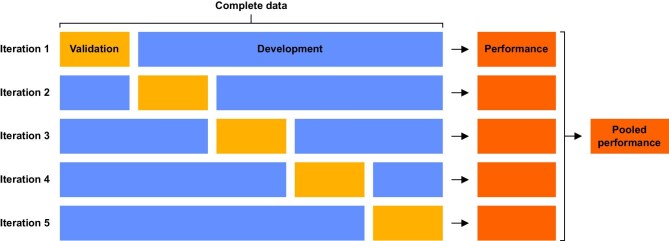
A visual representation of cross-validation. In 5-fold cross-validation, we split the data into five equal folds, of which four are used for model development and five are used for validation. After all five iterations, the final performance is determined by averaging the performance measure.

Fourth, prediction models often perform worse outside of their development population, as the model is finetuned to that population. Therefore, prediction models always require validation [24]. Validation methods lie on a spectrum: on one end lies apparent validation, which is the performance of the prediction model in the development population. On the other end lies external validation targeted at populations of interest [25], which says something about generalizability to that target population. If we do not perform a validation targeted at a new population of interest, we cannot reliably use the prediction model in that new population of interest. Some validation methods are closer to external validation (e.g. temporal and geographical validation) while others are closer to apparent validation, such as internal validation. Internal validation can for instance be used to assess reproducibility, with the best methods being bootstrapping and cross-validation. With bootstrapping, we randomly sample individuals with replacement (meaning the same individual can be drawn more than once) until we have a dataset of the same size as the original dataset. We redevelop the prediction model in this dataset and apply it to the original dataset. By doing this many times, we can estimate overfitting-corrected performance measures [26]. For cross-validation, we determine *k* folds (e.g. 5 folds, each containing 20% of the data). We then develop the model in *k* − 1 folds and validate it in the left-over fold, calculating our performance measures [24]. The final performance measures are obtained by averaging over all validations’ results (Fig. 2).

Importantly, regarding validation, we should remain aware that model validation is never finished. With the passing of time, models may suffer from calibration drift, for instance due to the underlying prevalence of disease changing [27]. Specifically and paradoxically, a well-performing model may help diminish the prevalence of the outcome, leading to poorer model performance in the future. Additionally, coefficients may not accurately represent the association between the predictor and the outcome in the future. For example, the association of diabetes mellitus with kidney failure may be reduced with the introduction of sodium-glucose cotransporter 2 inhibitors (SGLT2i) and the predictive value of diabetes mellitus for kidney failure may subsequently decrease.

Fifth, impact analyses should be performed before widespread implementation of a prediction model, to explore whether it has a positive impact on healthcare [28]. A prediction model might not make an impact for many reasons, such as a lack of uptake. Impact analyses help avoid implementation and maintenance of unimpactful prediction models and safeguard patients’ well-being by warding against harmful prediction models [28]. Impact analyses are most conclusive in the form of an impact cluster randomized controlled trial, but a combination of alternatives may also suffice [29]. Although this is the last principle we highlight, envisioning the intended impact already during conceptualization (the first principle) and collaborating with relevant stakeholders will help the model be impactful in its intended setting.

Sixth and last, for the implementation of a prediction model, applicable local legislation should be adhered to. For instance, given that prediction models aim to inform or even direct clinical practice, they would be considered medical devices and would be subject to the Medical Device Regulation in the European Union. Among others, this means that the model would require a conformity assessment prior to market authorization. A more extensive comment on the Medical Device Regulation and the European Framework for AI is available elsewhere in the *Clinical Kidney Journal* [3]. Legislation applicable to clinical prediction models also exists outside of the European Union [30].

## PART I: CONVENTIONAL STATISTICS

Prediction modelling using conventional statistics most commonly entails using regression techniques based on a prespecified linear predictor (i.e. the combination of regression coefficients and individual predictor values). The linear predictor is linked to an outcome based on the type of data (Box 1). Once we have a relevant outcome to be predicted with a clear time zero and suitable data, we can choose a suitable regression method.

Box 1. **How different regression models calculate individual predictions.**

Regression models relate the outcome to the predictors by using a linear predictor ($LP$), which is the sum of a constant term (the intercept) with the combination of the other regression coefficients (${\beta _j}$) and the respective individual predictor values (${x_{ij}}$) of individual $i$. An individual's $L{P_i}$ for a prediction model with $p$ predictors can be calculated as:

\begin{eqnarray*}
L{P_i} = \mathop \sum \limits_{j = 0}^p {\beta _j}{x_{ij}} = {\beta _0} + \,\,{\beta _1}{x_{i1}} + {\beta _2}{x_{i2}} + \ldots + {\beta _p}{x_{ip}}
\end{eqnarray*}

The $L{P_i}$ is used by regression formulas to arrive at the final individual prediction ${\hat y_i}$. Below, we showcase how different regression models achieve this.

Linear regression

\begin{eqnarray*}
{\hat y_i} = L{P_i}
\end{eqnarray*}

Logistic regression

\begin{eqnarray*}
{\hat y_i} = \,\,\frac{1}{{1 + \,\,{e^{ - \,\,( {L{P_i}} )}}}}
\end{eqnarray*}

Poisson regression

\begin{eqnarray*}
{\hat y_i} = \,\,{e^{L{P_i}}}
\end{eqnarray*}

Cox regression

\begin{eqnarray*}
{\hat y_i}( t ) = 1 - \,\,{S_0}{( t )^{{e^{L{P_i}}}}},\,\,{S_0}( t ) = \,\,{e^{ - {H_0}( t )}}
\end{eqnarray*}

where ${H_0}(t$) is the cumulative hazard function at time $t$ and ${\beta _0} = 0$ in the $L{P_i}$.

### Choosing a regression method

The regression method we use depends primarily on what we are trying to predict. If we are trying to predict a continuous outcome, such as estimated glomerular filtration rate (eGFR), we can use linear regression. However, we may instead be interested in predicting a binary outcome, such as an eGFR below 15 ml/min/1.73 m^2^ (which we might name kidney failure), in which case we would use a logistic regression model. Nonetheless, if we have a relatively long follow-up, we should take into account censoring, which would mean using a Cox regression model. Importantly, if an individual dies before kidney failure, we were unable to observe their time to kidney failure due to the competing risk of death. In the presence of competing risks, we would overestimate the predicted risk of kidney failure by using Cox regression [22, 31], but this could be alleviated by using a Fine–Gray regression model.

It may be clear that the different regression models each have their advantages, with more models being available for different situations, such as count data (e.g. the number of hospitalizations using Poisson regression) or dynamic prediction (e.g. updated predictions over time using joint modelling).

Additionally, different regression models can be used for the same goal: instead of logistic regression we can use probit regression, instead of joint models we can use landmarked Cox regression, and instead of Fine–Gray regression we can use multistate models.

### Dealing with nonlinearity

Besides other assumptions, regression models commonly assume a linear association of the predictors with the outcome (or a specified transformation of the outcome, such as the logit for logistic regression). However, this is not always the case. For instance, body mass index (BMI) often has a U-shaped association with clinical outcomes: a low BMI and a high BMI tend to be harmful while a moderate BMI tends to be healthy.

**Figure 3:**
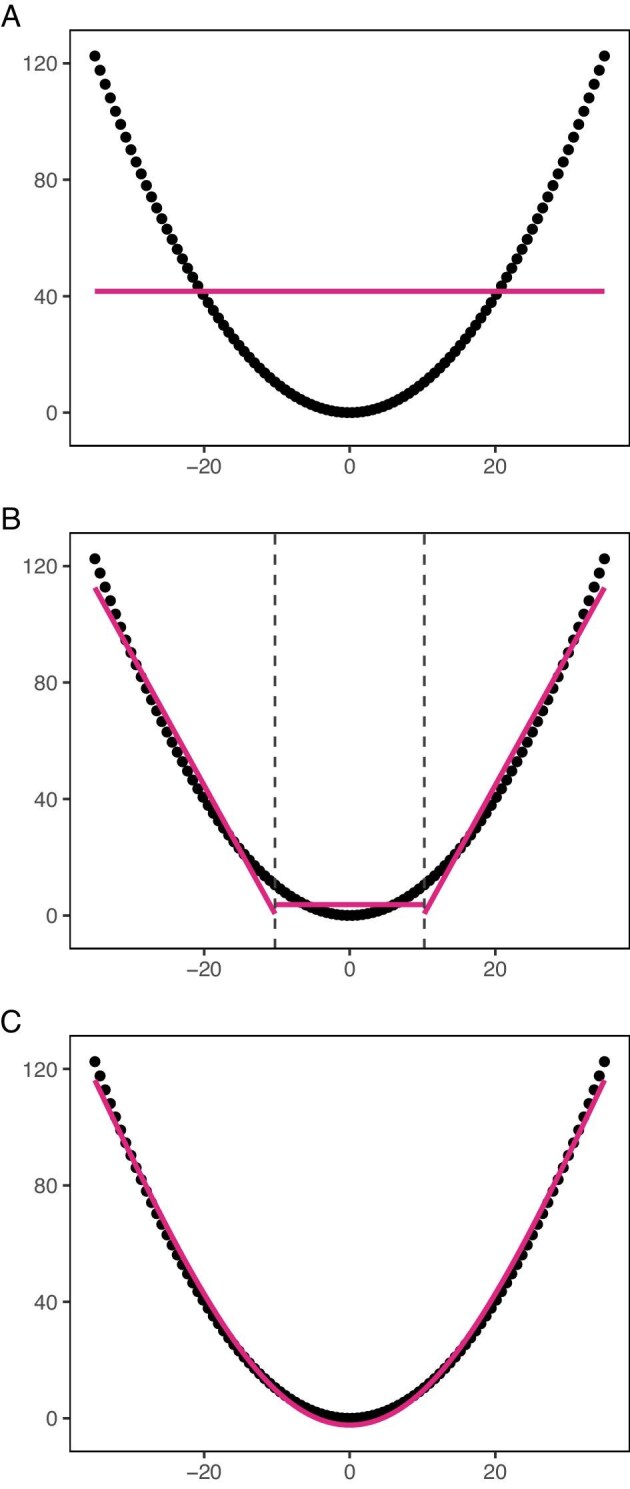
A visual representation of how splines work. The predictor would have no predictive value for the outcome if modelled linearly (**a**). However, we can use splines to transform the predictor. Intuitively, a spline cuts up the nonlinear association in smaller linear associations that are modelled separately, defined at cut-off points called knots (**b**). The spline function results in smooth transitions at the ends of the separate sections to join the line together (**c**). This will result in accurately modelling the relationship between the predictor and the outcome, as captured in multiple coefficients. In this example, we used a natural cubic spline with two prespecified knots at −10 and 10 for (c). Simulated data.

Although many solutions exist, such as transformations and polynomials, splines offer a simple and effective solution by breaking up a predictor's nonlinear association with the outcome in separate pieces, which are then all modelled linearly (Fig. 3). Each piece gets its own regression coefficient and whether that regression coefficient contributes to the final prediction depends on an individual's value for the predictor [32]. An extensive discussion of dealing with nonlinearity is available elsewhere [33].

### Developing and validating the prediction model

To develop the prediction model, we need to estimate the regression coefficients of the included predictors. These are used to calculate the final predictions and are most often estimated using a maximum likelihood estimator (that determines which values of the regression coefficients fit the data best).

If we developed our prediction model in a small dataset, we might risk overfitting. In such a case, we could shrink the regression coefficients, for instance using bootstrapping [34], which makes the model less overfitted and more generalizable. Some regression methods exist that penalize regression coefficients by default. Ridge regression shrinks regression coefficients (albeit never to zero) as does least absolute shrinkage and selection operator (LASSO) regression (which can shrink regression coefficients to zero, effectively removing those predictors from the prediction model). Their penalizations can also be combined (and balanced) using elastic net regression.

### Presentation of the final model

The final prediction model will be a formula that can easily be implemented in online calculators and which can be calculated by hand (if not too many predictors are present). In the final model, the regression coefficient of each predictor can be used to get an indication of the relative contribution of that predictor to the final prediction, taking into account their range of possible values. Although these regression coefficients cannot be interpreted as a causal effect (Box 2), they do give us information on which predictors influenced the final prediction most. The final prediction model should be reported according to the Transparent Reporting of a multivariable prediction model for Individual Prognosis Or Diagnosis + Artificial Intelligence (TRIPOD + AI) statement [35], which ensures all required information to assess and use the prediction model is available to the reader.

Box 2. **Causal statements based on prediction models**

When we predict the risk of an outcome (or any other prediction) for an individual, we may be inclined to say something about how that prediction would change if an individual's value for a predictor were to change. For instance, we might be inclined to say that an individual's risk of kidney failure as predicted by the KFRE decreases if we decrease their albumin-creatinine ratio. However, the newly estimated risk with the decreased albumin-creatinine ratio is probably incorrect for three main reasons.

First, the prediction is based on individuals who are similar in their values for predictors to our individual of interest. If we change the value of a predictor, the prediction is based on individuals who are not so similar to our individual and thus the risk may not be accurate.

Second, a change in a predictor's value is often not instantaneous. Changing a predictor's value may take time during which risk may still accumulate.

Third, the predictor might not be the cause of the change in the predicted outcome, because a prediction model often does not explicitly account for the underlying causal structure between predictor and outcome.

Although techniques exist to make such causal claims (counterfactual prediction), most prediction models do not allow causal statements for these reasons.

## PART II: MACHINE LEARNING

Similar to conventional regression models, supervised ML techniques (i.e. techniques using a prespecified outcome) are used for prediction modelling and learn from data. Although the learning process is often more computationally involved, ML techniques are still a combination of mathematical formulas [36, 37, 38]. ML techniques require less manual specification, as the final model structure is learned from the data. This makes it easier to obtain complex model structures, which generally improves predictive performance in complex clinical scenarios. However, this also makes the final prediction model harder to understand. Common techniques include *k*-nearest neighbour (*k-*NN), support vector machines (SVMs), random forests (RFs), and ANNs. Analogous to prediction modelling using conventional statistical techniques, once we have a relevant outcome with a clear time zero and suitable data, we can choose a suitable ML technique.

### Choosing an ML technique

In Part I, we have already discussed one of the least computationally involved techniques that is also often considered ML: regression modelling [39]. This is not a surprise as regression models also ‘learn’ the value of the regression coefficients based on the data.

**Figure 4:**
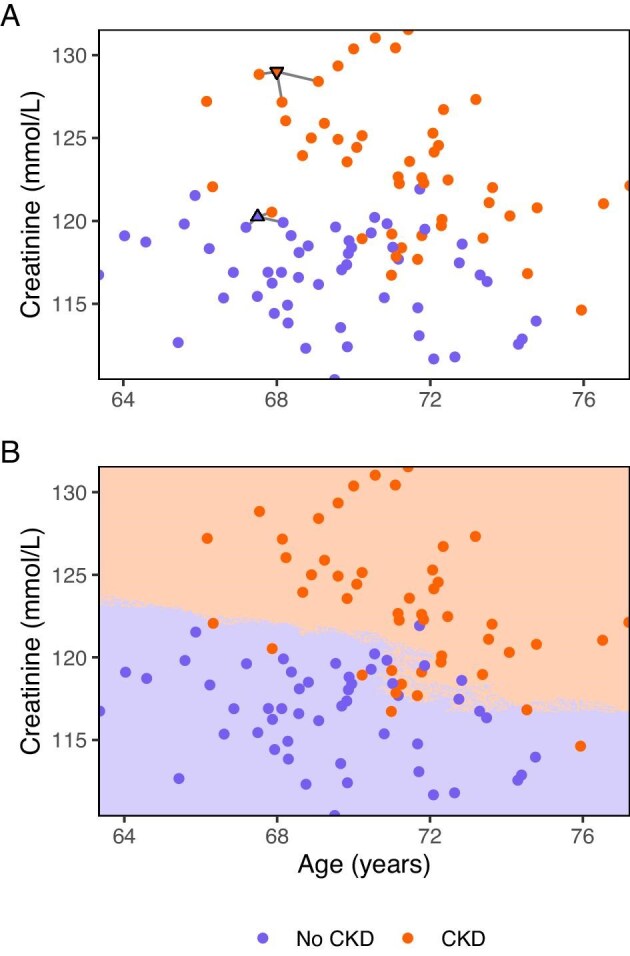
Classification using *k*-nearest neighbour. The dots are coloured according to the observed outcome, while the shaded areas represent the predicted outcome. To classify individuals, *k*-nearest neighbour, looks at the *k*-nearest neighbours (i.e. most similar observations). By taking a majority vote of their classification (outcome or no outcome), the new observation is classified. As an example, we used 3-nearest neighbours to classify two new observations (**a**). The down-facing triangle was nearest to three individuals with CKD and therefore classified as such. The up-facing triangle was nearest to one individual with CKD and two without, therefore being classified as no CKD based on the majority vote. Because this is a non-parametric approach, the classification is not bound by underlying distributions or linearity assumptions (**a**). Simulated data.

We can also use *k*-NN, in which predictions are made based on whether the *k-*nearest neighbours (i.e. most similar individuals) in the development data did or did not develop the outcome (Fig. 4). For continuous outcomes, the prediction is based on the observed value of the *k*-nearest neighbours. Further details for *k*-NN are available elsewhere [40].

**Figure 5:**
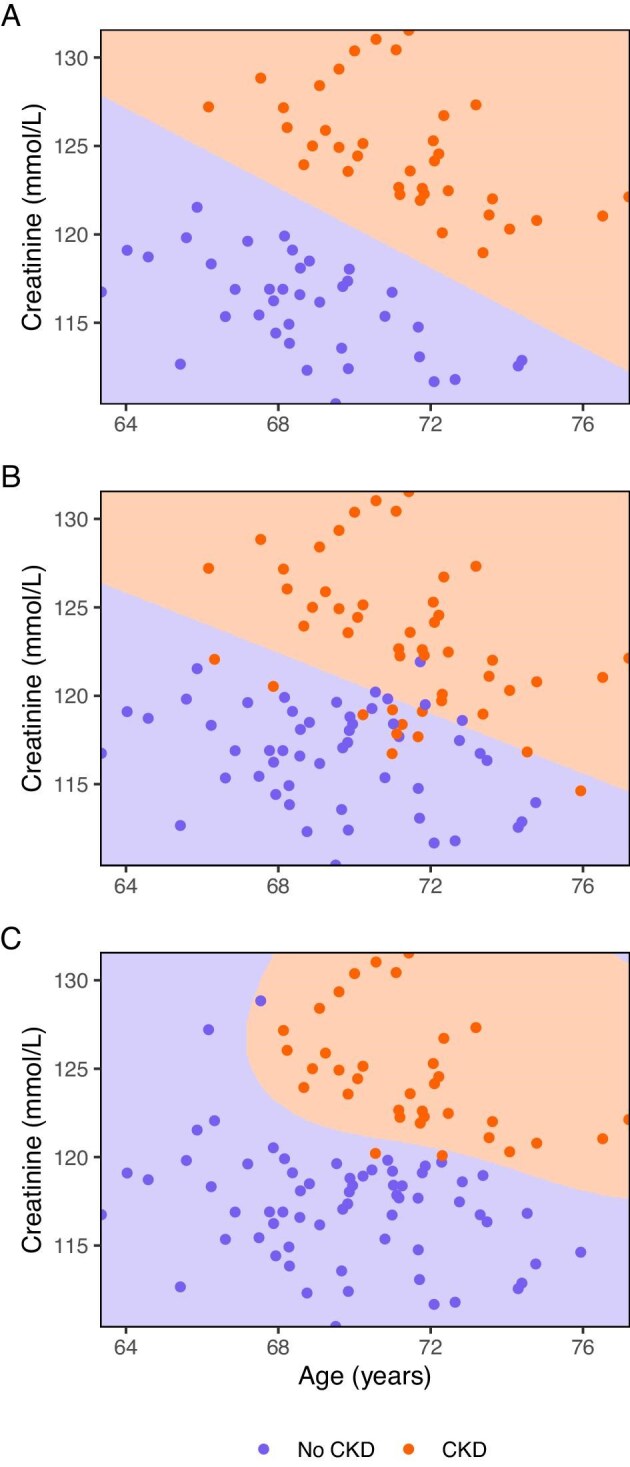
Classification using a support vector machine. The dots are coloured according to the observed outcome, while the shaded areas represent the predicted outcome. The support vector machine aims to find a linear boundary line (called hyperplane in higher-dimensional spaces) between individuals with and without the outcome that creates the largest distance between the two groups (**a**). If there is no clear distinction due to overlap, the support vector machine can still find a linear boundary plane by flexibly handling the observations that would overlap the boundary line (**b**). In the case that an ideal boundary line would not be linear, the support vector machine can find the linear boundary plane in a higher dimension using the kernel trick. The boundary line would be linear in that higher dimension but not *per se* in the original dimension (**c**). Simulated data.

Alternatively, using SVMs we might predict a binary outcome by finding a linear boundary that separates the individuals with or without a particular outcome by the largest distance (Fig. 5). Because a linear boundary might not always be identified in a two-dimensional plane, SVMs use a so-called kernel trick to increase the dimensionality of the plane. In the higher (to infinite) dimensional plane, the SVM can find the ideal linear boundary (which would be called a line in a two-dimensional space, a plane in a three-dimensional space, and a hyperplane in a higher-dimensional space). SVMs can also be extended to handle right-censored data, similar to Cox regression [41]. A detailed explanation of SVMs can be found elsewhere [42].

**Figure 6:**
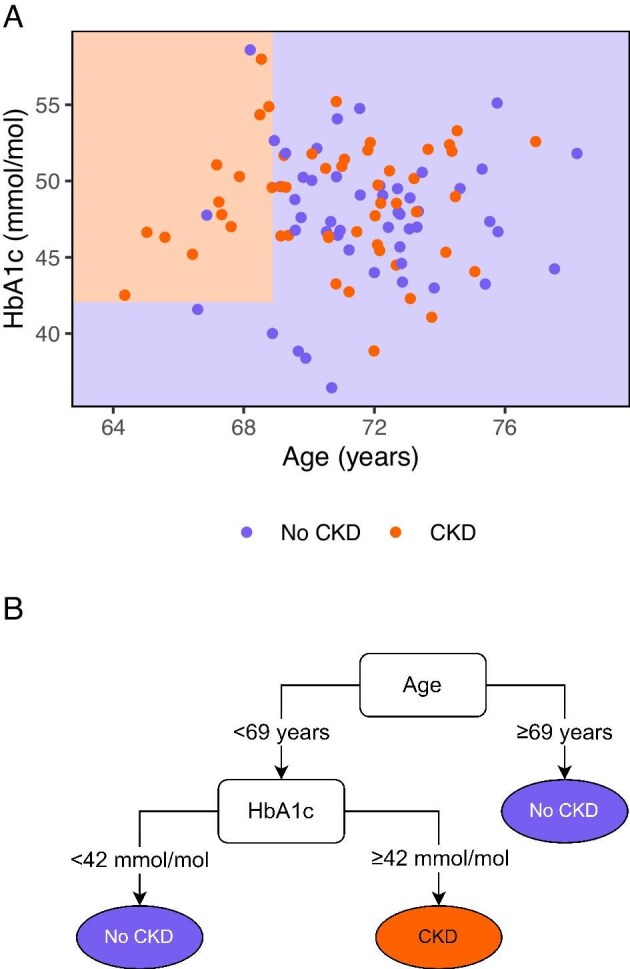
A decision tree underlying RFs. The dots are coloured according to the observed outcome, while the shaded areas represent the predicted outcome. RFs are based on many decision trees that all classify individuals into outcome or no outcome based on a randomly selected set of predictors (which in this example was capped to a maximum of two). This decision tree does not require high performance for the final random forest to have a high performance (**a**). The decision tree can also be portrayed as an actual tree (**b**). The random forest combines the classification of all trees into the probability of an outcome. Simulated data.

Ensemble learning is a subtype of ML, in which multiple base models with relatively poor performance (also known as. weak learners) are combined into a single better performing model. RFs are such an ensemble method, in which decision trees are the weak learners (Fig. 6). The decision trees are developed on bootstrapped data and then combined (a procedure that can be applied to many techniques, called bootstrap aggregating or bagging by portmanteau), leading to a more generalizable model without losing performance. Additionally, each decision tree uses a random subset of predictors to avoid correlations between decision trees. The final prediction is based on the average of the predictions of all underlying decision trees. An extension for survival data is also available for RFs as random survival forests [43]. An in-depth explanation of decision trees and RFs is available elsewhere [44].

Another ensemble technique that uses decision trees is gradient boosting. Gradient boosting is a boosting technique (i.e. a technique in which weak learners are iteratively improved, focusing on the errors of the previous weak learner) that adjusts weak learners in the direction that minimizes error the most per iteration (which is called gradient descent). Gradient boosting can also be based on other weak learners, including many regression models, such as logistic regression and Cox regression. A more in-depth guide is available elsewhere [45].

ANNs are a ML technique that can become highly computationally involved, aiming to model an outcome (continuous, binary, right-censored etc.) similar to how biological neurons function. ANNs can exist of multiple layers of nodes (i.e. artificial neurons), where the first layer is an input layer, the last layer is an output layer, and hidden layers are in-between. Inputs, such as an individual's predictors’ values, travel from the input layer through the hidden layers, where nonlinear activation functions transform the inputs at each node, to the output layer. The output layer transforms the input into the prediction of interest. Although ANNs can predict using tabular data, they are particularly useful when using non-tabular predictors, such as images. A primer into ANNs is available in a paper by Kriegeskorte and Golan [46].

These techniques were all used in a study by Güven *et al.* where they aimed to predict adverse kidney events related to renin-angiotensin-system inhibitors [47]. Although their models showed high discrimination, calibration was not shown and due to a relatively low sample size (*n* = 409 with 50 events), most models were probably overfitted.

If we wanted to select a specific ML technique, different aspects would play a role: the type of outcome to be predicted (e.g. continuous, binary, survival), the degree of nonlinearity between the predictors and the outcome, the degree to which model interpretability is important (e.g. if used to inform a patient, the patient might be interested in how the model arrives at the final prediction, requiring an interpretable model), and the size of the available data. For instance, *k*-NN is more interpretable than RFs and requires less data, but may not model complex relationships as well. ANNs can model complex relationships better, but require an abundance of data. Additionally, ANNs perform well with high-dimensional data (e.g. combining tabular predictors and images), but may be outperformed by gradient boosting if predictors are tabular. Last, software is unlikely to be held responsible when a mistake occurs. We should take this into account when choosing for a model. Some require less manual input, which means certain decisions regarding the model are made that we do not directly control, but for which we are responsible.

### Dealing with nonlinearity

Different ML techniques deal with nonlinear relations differently. Some ML techniques are inherently non-parametric, such as the decision trees underlying gradient boosting and RFs, and *k*-NN. The model architecture may also allow nonlinearity. For instance, SVMs use the kernel trick to approach the prediction in a higher dimension where a linear boundary (the hyperplane) can be found. In gradient boosting, we can use splines (similar to regression methods) or radial functions and ANNs apply nonlinear activation functions to their inputs.

Thus, ML generally uses a data-driven approach to deal with nonlinearity, while in conventional statistics more manual specification by the researcher is necessary. However, manual specification does allow the researcher to have more control over model development, avoiding overfitting by limiting the number of transformations, and offers insight in how the model works.

### Developing the prediction model

ML models may become overly complex, resulting in overfitting. Model complexity can be managed by hyperparameter specification, such as the depth and number of decision trees in RFs, the number of layers and neurons in ANNs, and the number of boosting iterations for gradient boosting. Hyperparameters influence how the model is trained and are usually learned from the data. To this end, development data are partitioned into two parts: a training set (for initial model development) and a tuning set (often called a validation set) (Table 1). Models with different hyperparameter configurations are developed on the training set and subsequently applied to the tuning set. The optimal parameters within the model are determined by a loss function that aims to maximally reduce error. The model with the best performance metric (e.g. the *C*-statistic) will determine the hyperparameter configuration used to train the final model on the full development data. Afterwards, the model might be validated in a test set. It is noteworthy that in ML the data are commonly randomly split into training, validation, and test datasets. Because the test dataset is then highly similar to the training and validation data, the performance assessment in the test set classifies as internal instead of external validation [24].

### Presentation of the final model

Whereas the final prediction model developed using conventional statistics gives a relatively simple formula, this is often not the case for prediction models developed using ML techniques. To allow others to use the prediction models (and perhaps validate them), a method to interact with and apply the prediction model should be made available. This could simply mean sharing the model object to make predictions within the programming language in which it was developed (e.g. R or Python). An alternative would be a (web-based) application to calculate individual risks, which is more accessible than using programming language-specific model objects.

Determining how predictors influence the final prediction is often tougher for ML techniques, leading to a ‘black box’ phenomenon. Although this is a criticized limitation of ML techniques, there is a field (explainable ML) that focuses on explaining the black box [48, 49]. One general method is the variable importance score, which ranks predictors based on their influence in the prediction model [50]. A more detailed method is Shapley additive explanations (SHAP). SHAP can give insight in the contribution of a specific predictor to a specific individual's prediction, as well as the relative influence of a specific predictor on the full prediction model [51].

As with conventional statistics, the final prediction model should be reported according to the TRIPOD + AI statement [35], to ensure the required information for assessment and use of the prediction model is available to the reader.

## THE SUM OF THE PARTS

Certain differences exist in developing prediction models with conventional statistics and ML, although to a certain extent this is just different vocabulary with the same meaning (Table 1) [14].

Techniques might come across as different, but still share many similarities. For instance, an ANN without a hidden layer and with a single neuron containing a logistic activation function in the output layer is equivalent to logistic regression. Additionally, models underlying gradient boosting can be the same models used in conventional statistics, and ridge/LASSO regression also require hyperparameter tuning (for the regulation parameter *λ*). Finally, some ML techniques may use loss functions akin to maximum likelihood estimation, and both conventional statistics and ML often use bootstrapping and cross-validation.

At the same time, conventional statistics and ML face the same obstacles. Overfitting impedes generalizability and may occur for all techniques. Moreover, missing data are often present in multiple predictors. Although many models can function in the presence of missing data, their estimation might not be reliable, especially if the missingness is informative for the outcome (e.g. sicker individuals are less likely to attend study visits and more likely to die) [52]. Last, validation studies and impact analyses remain scarce.

**Figure 7:**
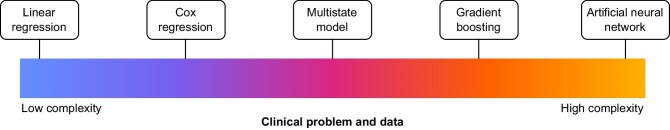
The spectrum of clinical problem and data complexity and where techniques used in conventional statistics and ML fall. The spectrum ranges from data and clinical problems with low complexity (e.g. single timepoint, normally distributed continuous outcome, few predictors) to high complexity (e.g. multiple dynamic outcomes, large number of predictors). We show at what complexity of the clinical problem and data different techniques would best be used.

As such, we prefer to think of conventional statistics and ML as a single field with the same goal [14]. The different techniques are complementary and lie on a spectrum (Fig. 7, Box 3): on the left-hand side, we have relatively non-complex clinical problems with low-dimensional data and clear predictors. In such settings, conventional statistics provide computationally easy, interpretable, and transparent prediction models. For these situations, we do not need an ‘overkill’ of complex models: we can use non-complex models for non-complex situations and leave complex models for situations with higher-dimensional data, more complex relationships, and many available predictors [53]. In these complex situations, on the right side of the spectrum, ML techniques are ideal for developing well-performing prediction models, although it is important to mention that they require much more data than techniques used in conventional statistics.

Box 3. **The bias-variance trade-off**

We can also view the models on this spectrum from a bias-variance trade-off perspective. If presented with high-dimensional complex data, models on the left side of the spectrum tend to have more bias (i.e. predictions are less accurate), but less variance (i.e. models are less overfitted and more generalizable). These models can reduce bias by using splines and interaction terms. Conversely, models on the right side of the spectrum tend to be less biased (i.e. predictions are on average more accurate), but also have more variance between different samples from the same population (i.e. models are more overfitted and less generalizable). These models aim to reduce variance by limiting complexity and combining multiple smaller models (e.g. using ensemble learning).

Per clinical problem and complexity of the available data, we can determine the techniques that best suit our situation (as described in Parts I and II) and use these to develop multiple prediction models. From those, we can select the best performing prediction model and move forward with it to targeted external validation and impact analyses. The comparison should not be about conventional statistics vs. ML, but about what technique best suits our current clinical problem and available data.

## THE WHOLE

Combined, conventional statistics and ML can help us develop the best prediction models for patients and healthcare providers. However, we may gain even more by integrating them.

An important topic in ML is algorithmic fairness. This topic focuses on how systematically biased data leads to systematically biased predictions and how to correct this. For instance, if chronic kidney disease (CKD) was underdiagnosed in females (thus actually present but not captured in the data), the prediction model would systematically underpredict the risk of CKD in females. Consequently, females could have impaired access to necessary resources in preventing further kidney damage. Algorithmic bias should first and foremost be prevented by an accessible and equitable health care system, but may be temporarily alleviated by modelling decisions, such as which outcome is predicted (although this may not fully resolve the problem) [54]. Despite its importance, algorithmic fairness is not currently a big topic in conventional statistics. Nonetheless, to develop fair prediction models and prevent systematic bias as a result of prediction models, we should be paying attention to how the underlying data are generated, remain open to sharing our work and relevant details on the development of the model, allow our peers to critically appraise our models, and keep monitoring for systematic bias even after implementation.

After a prediction model is developed, reporting should be clear enough for the model to be used by others (if this is intended) and include key components (i.e. intercept and regression coefficients for regression models or model objects plus code for ML techniques). However, the reporting of ML models could be improved by following guidelines originally developed for conventional statistics (e.g. TRIPOD + AI) [35, 55].

By working together, conventional statistics and ML may further intertwine as disciplines, focusing on developing, validating, and implementing the best prediction models. To this end, we can compare different models from both disciplines to get the best predictions for specific clinical problems. However, we must refrain from detrimental comparisons, such as conventional statistics vs. ML. The focus should in the end be on improving healthcare for the patient, instead of attempting to prove superiority of one scientific discipline over the other.

## Data Availability

All data underlying the examples were simulated. The R code used to generate this data and the accompanying figures are available at https://github.com/rjjanse.
